# Emphysematous cystitis as a potential marker of severe Crohn's disease

**DOI:** 10.1186/s12876-022-02253-6

**Published:** 2022-04-11

**Authors:** S. M. Mahmudul Hasan, Baljinder S. Salh

**Affiliations:** 1 Health Sciences Centre, Memorial University of Newfoundland, 300 Prince Phillip Drive, St. John’s, NL A1B 3V6 Canada; 2 Division of Gastroenterology, University of British Columbia, Gordon & Leslie Diamond Health Care Centre, 2775 Laurel Street, Vancouver, BC V5Z 1M9 Canada

**Keywords:** Crohn’s colitis, Emphysematous cystitis, Colovesical fistula, Case report

## Abstract

**Background:**

Emphysematous cystitis (EC) is characterized by the presence of air within the bladder wall, often a complication of urinary tract infection (UTI) by gas-producing organisms. However, EC has also been reported in the setting of infectious colitis suggesting an alternate etiology. We report a rare case of EC in the setting of severe Crohn’s colitis with no clinical evidence of UTI.

**Case presentation:**

A 43-year old female presented with a 2-month history of bloody diarrhea consisting of 8–12 bowel movements a day, weight loss of 10 kg and peripheral edema. She also had multiple ulcerated lesions on her abdominal wall and in the perianal region. Initial CT scan was significant for pancolitis, anasarca and EC. The follow-up CT cystogram, flexible cystoscopy and pelvic MRI confirmed the diagnosis of EC and ruled out any fistulous tracts in the pelvis including enterovesical/colovesical fistula. The patient did not report any urinary symptoms and the urinalysis was within normal limits. An extensive infectious workup was negative. Despite the paucity of infectious findings, the EC was empirically treated with an intravenous third-generation cephalosporin. Colonoscopy was significant for multiple ulcerated and hyperemic areas with pseudopolyps all throughout the right, transverse and left colon. Biopsies confirmed Crohn’s colitis with no evidence of granulomata or dysplasia. Immunohistochemistry was negative for CMV. The perianal and abdominal wall lesions were suspected to be pyoderma gangrenosum although biopsies were equivocal. The colitis was initially treated with intravenous steroids followed by biologic therapy with Infliximab. Despite appropriate escalation of therapies, the patient developed colonic perforation requiring subtotal colectomy.

**Conclusion:**

This is a rare case of EC in a patient with severe Crohn’s colitis. There was no evidence of urinary tract infection or fistulising disease. According to our review, this is the first reported incident of EC in a patient with inflammatory bowel disease without any prior intra-abdominal surgeries. While active Crohn’s disease alone is a critical illness, we conclude that concomitant EC may be a poor prognostic factor.

## Background

Emphysematous cystitis (EC) is often a complicated urinary tract infection (UTI) that presents with severe clinical sequelae. It is defined by the presence of gas within the bladder wall, which is identified through imaging studies [1, 2]. Risk factors for developing EC include advanced age, poorly controlled diabetes, immunosuppression, chronic UTIs and urinary tract outlet obstruction [3, 4]. In addition to an ascending infection by gas-producing organisms, EC can also result from abdominal or pelvic instrumentation, fistula to a hollow viscus and tissue infarction with necrosis [5].

There have been multiple reports of EC in the setting of infectious colitis, both with and without any microbiological evidence of a concurrent UTI [6, 7]. Acute colonic inflammation from diverticulitis has also been associated with EC, some cases of which were in the context of fistula formation between the colonic diverticula and the bladder [8]. In the absence of obvious colovesical fistula, the connection between colonic inflammation and EC is poorly understood.

Crohn’s disease is a relapsing systemic inflammatory disease that may affect any segment of the gastrointestinal tract and give rise to complications like fibrosis, stricturing and fistula formation [9]. Crohn’s colitis presents as transmural and segmental inflammation that is restricted within the colon, although these patients may still exhibit features of extra-intestinal manifestations and perianal disease [10]. We report the first case of EC in the setting of severe Crohn’s colitis with no evidence of UTI or fistulating disease. This highlights EC as a potential unappreciated sign of severe Crohn’s disease that is worthy of further investigation.

## Case presentation

A 43 year-old female presented to a community hospital in Yukon, Canada with a 2-month history of bloody diarrhea. On admission a plain radiograph identified a large mediastinal mass. She was subsequently transferred to Vancouver General Hospital (VGH) under the care of Thoracic Surgery. Prior to her transfer she received oral steroids and 5-ASA formulations for 2 days as her initial stool cultures were negative and a diagnosis of inflammatory bowel disease (IBD) was suspected.

The patient was an immigrant from Taiwan and had previous episodes of bloody diarrhea with associated abdominal discomfort and fatigue. Her prior symptoms never lasted for more than a month and the most recent episode was 18 months ago when she was still living in Taiwan. She had no previous investigation for her symptoms. She presented to the hospital due to the fact that her symptoms had persisted for 2 months over which she lost 10 kg and developed significant peripheral edema. On presentation she was having 8–12 episodes of bloody diarrhea daily. The laboratory investigations on admission are highlighted in Table 1. This was significant for normocytic anemia, hypoalbuminemia, elevated ferritin and elevated C-reactive protein. The patient also had multiple round and shallow ulcers with a punched-out appearance on her abdomen. Furthermore, she had a large perianal ulcer, which had developed over the preceding two weeks. She did not have any history of ocular diseases, arthralgia, or any other history of dermatological symptoms.

**Table 1:** Summary of laboratory investigations on admission Hospital with appropriate reference ranges

Parameters	Reference range
*Hematology*	
Hemoglobin—81 g/L	120–155 g/L
MCV—91fL	82–98 fL
Platelets—651 × 10^9^/L	150–400 × 10^9^/L
Leukocytes—17.1 × 10^9^/L	4–11 × 10^9^/L
*Coagulation studies*	
INR—1.2	0.9–1.2
PTT—33 s	25–38 s
*Electrolytes and metabolites*	
Sodium—139 mmol/L	135–145 mmol/L
Potassium – 3.1 mmol/L	3.5–5.0 mmol/L
Chloride—108 mmol/L	95–107 mmol/L
Total CO_2_—23 mmol/L	22–31 mmol/L
Urea—4.2 mmol/L	2.0–8.2 mmol/L
Creatinine—36 μmol/L	40–95 μmol/L
Glucose—6.9 mmol/L	3.6–11 mmol/L
Calcium (ionized)—1.15 mmol/L	1.1–1.3 mmol/L
Phosphate—0.93 mmol/L	0.8–1.45 mmol/L
Magnesium—0.75 mmol/L	0.7–1.1 mmol/L
*Enzyme and protein chemistry*	
Amylase—14 U/L	35–90 U/L
Lipase—89 U/L	0–393 U/L
AST—16 U/L	10–38 U/L
ALT—12 U/L	10–45 U/L
ALP—147 U/L	30–135 U/L
Bilirubin (total)—5 μmol/L	< 20 μmol/L
Albumin—11 g/L	34–50 g/L
C-reactive protein—40.8 mg/L	< 3.1 mg/L
Lactate Dehydrogenase—151 U/L	90–240 U/L
Ferritin—1224 μg/L	15–130 μg/L

**Fig. 1:**
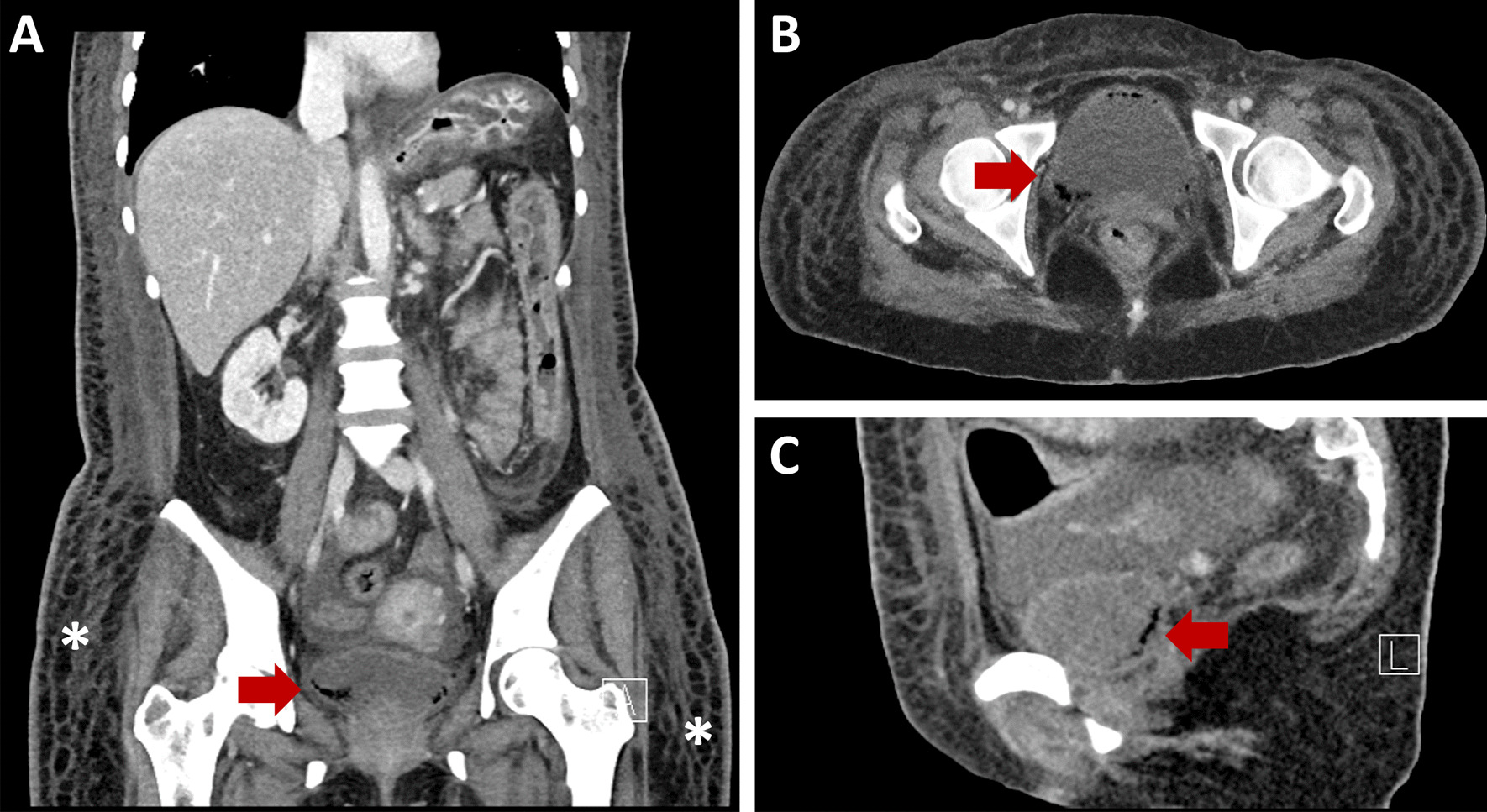
Non-contrast enhanced CT scan of the abdomen and pelvis showing diffuse anasarca (*) and emphysematous cystitis (red arrow) in the **a** coronal plane. Emphysematous cystitis is also shown in the **b** axial and **c** sagittal planes

In terms of diagnostic imaging the patient had a contrast-enhanced CT scan of her chest, abdomen and pelvis on arrival to VGH. There was a large heterogeneous soft tissue mass arising from the inferior aspect of the left thyroid lobe that measured 6.5 × 5.7 × 8.2 cm (transverse, antero-posterior, craniocaudal dimensions respectively). This mass extended inferiorly to the level of the carina and displaced the great vessels anteriorly without restricting blood flow. The abdomen and pelvic scans identified circumferential mural thickening of the entire colon with multiple pseudopolyps. The small bowel appeared unremarkable. There was significant anasarca and small volume ascites. The patient also had intramural gas foci within the bladder, a pathognomonic feature of EC (Fig. 1a–c). There was no bladder wall thickening and the urinary system had symmetrical enhancement with no obvious mass or obstruction. The patient did not report any urinary symptoms and both initial and subsequent urinalyses were within normal limits. Infectious workup including urine and blood cultures, stool ova and parasite, stool *C. difficile* PCR, TB-IGRA, Hep A, Hep B, Hep C and HIV serologies were all negative. The patient was consulted to the Urology service who agreed with the diagnosis of EC and arranged a flexible cystoscopy and a CT cystogram. The cystoscopy revealed patchy areas of submucosal hemorrhage throughout the posterior bladder wall. There was no evidence of any fistulous tract on direct visualization or on the CT cystogram. Lastly, a pelvic MRI was completed which definitively ruled out any fistulating disease. The EC was empirically treated with an intravenous (IV) third-generation cephalosporin for five days despite no obvious infectious etiology.

**Fig. 2:**
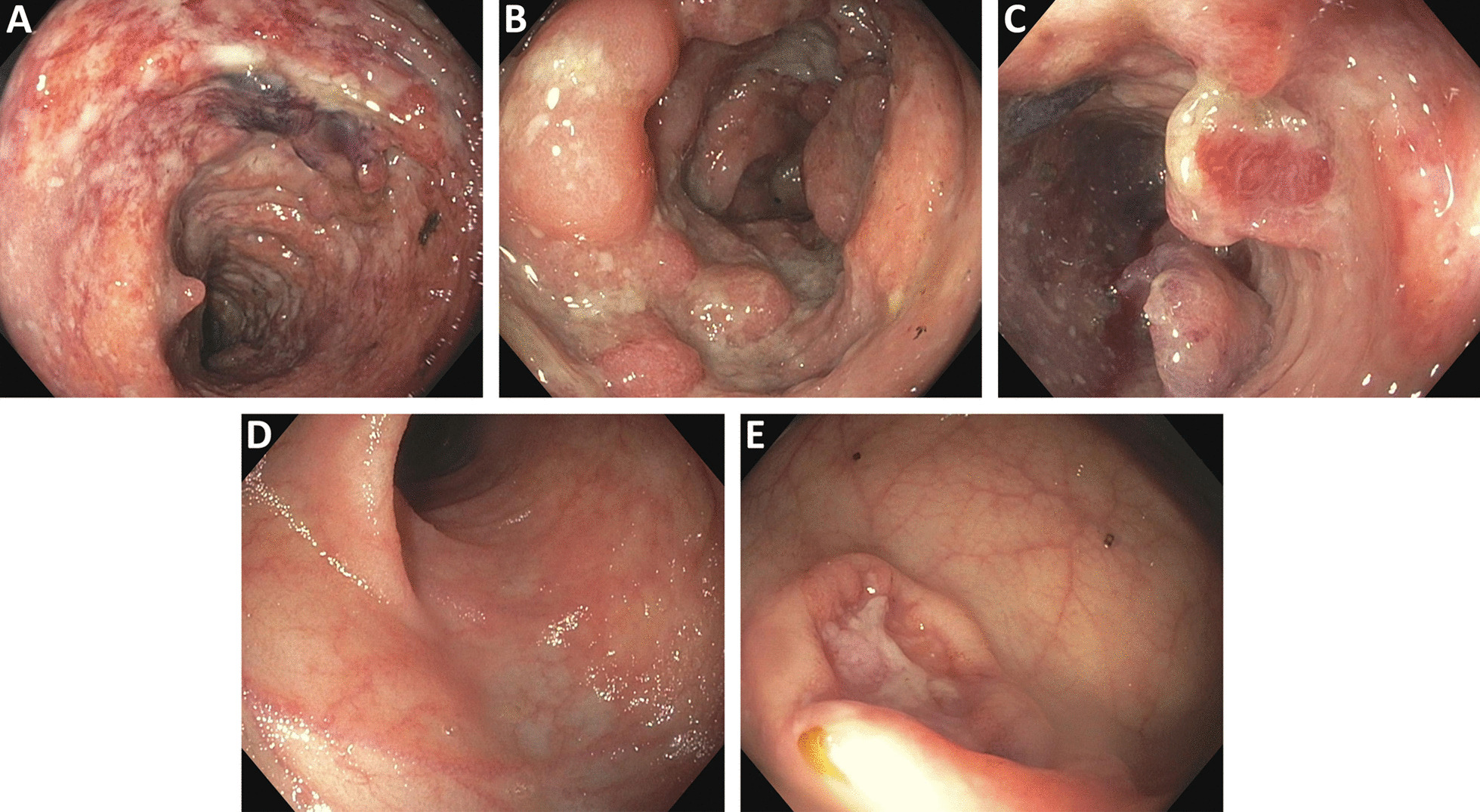
Significant ulcerated and hyperemic areas throughout the **a** right and **b** transverse colon with **c** inflammatory pseudopolyps. Less marked ulceration of the **d** left colon and **e** rectum

While the EC was investigated the patient also underwent a colonoscopy. This was significant for hyperemic and ulcerated areas with pseudopolyps throughout the right and transverse colon (Fig. 2a–c). There was less marked ulceration of the rectum and the left colon, which made this suspicious for colonic Crohn’s disease (Fig. 2d, e). The terminal ileum appeared normal. Colonic biopsies confirmed the diagnosis of Crohn’s colitis with no evidence of granulomata or dysplasia. CMV immunohistochemistry was negative. Dermatology service was consulted for the skin lesions. Biopsies of the abdominal wall ulcers showed non-specific inflammatory changes with neutrophilic infiltrates. The perianal ulcer also showed non-specific inflammatory changes without granulomatous evolution, which was equivocal for pyoderma gangrenosum. The biopsy samples did not show any features of infection or malignancy.

The patient was started on IV Methylprednisone while the known mediastinal mass was being investigated. An endobronchial ultrasound guided biopsy of the left paratracheal mass was diagnostic for goiter with no features of malignancy. Following this result, the patient was started on Infliximab induction therapy. Throughout her admission, she also received multiple units of packed red blood cells and IV albumin. With the initiation of Infliximab and IV steroids, the patient had steady improvement of her bloody diarrhea and the cutaneous lesions resolved. She was repatriated to Yukon from VGH on a tapering dose of oral prednisone and maintenance Infliximab infusions. However, following the transfer the patient developed a colonic perforation requiring subtotal colectomy with end ileostomy. Her post-operative course was further complicated by a stomal obstruction and interloop abscesses requiring multiple laparotomies and surgical revisions.

On the most recent follow-up assessment the patient had no known recurrence of Crohn’s disease including any perianal disease, and as a result she was not on any immunosuppressive therapies. She was followed up until about three years from her initial presentation with EC and colitis. She had normal stoma function and was being considered for an ileorectal anastomosis. She did not have any intervention for her goitre, and it had not caused any further complications.

## Discussion and conclusions

We report the first case of EC in the setting of severe Crohn’s colitis with no clinical evidence of a UTI. Our patient did not have any of the known risk factors for EC such as diabetes, severe immunodeficiency, systemic infection, hematuria or intra-abdominal instrumentation [1, 3, 11]. While immunological dysfunction is a significant component of Crohn’s disease, it does not make patients immunocompromised and susceptible to infections unless they are on immunosuppressive therapies [12].

CT scan is considered the ideal diagnostic modality for EC. This can characterize the extent of the disease beyond the bladder into the pelvicalyceal systems and renal parenchyma [13, 14]. Since colovesical fistula is a known complication of Crohn’s colitis [15] we completed a comprehensive workup to rule out any fistulating disease. In collaboration with Urology, we confirmed the diagnosis of EC and collectively ruled out any fistulous tract within the pelvis.

A literature review on PubMed and the Cochrane Library did not identify any documented association between IBD and EC. There is one reported incident of EC in an elderly patient following a total colectomy for ulcerative colitis [16]. Since abdominal instrumentation can be a precipitating factor for EC, it is difficult to interpret this connection. Despite the paucity of evidence for IBD, there is well established association between other types of acute colonic inflammation and EC. Acute diverticulitis can create colovesical fistulas, some of which may present as EC [8, 17–19]. Colon cancer arising within a colonic diverticulum can also create such colovesical fistula [20, 21]. Invasive colon cancer has also been known to have emphysematous presentation within the genitourinary system [22]. With multiple imaging modalities, direct visualization and pathological examination, we have definitively ruled out these more common etiologies for EC in our patient.

After a thorough literature review, we identified two reports of EC in the setting of infectious colitis [6, 7]. Both of these patients had a history of type 2 diabetes mellitus, presented with *C. difficile* colitis, and had evidence of EC on CT scan. However, only one of them had a concomitant UTI [6]. The presence of EC without any evidence of a UTI is not very common. In the absence of urologic infections, treatment of EC is best directed at the underlying disease process [1]. Under such circumstances the EC should be considered a clinical sign and not the primary disease process [7].

The association between colonic inflammation and EC is poorly understood. One possible explanation is bacterial translocation within the abdomen from the disruption of the mucosal barriers secondary to chronic inflammation [6, 23]. A similar mechanism is seen in severe IBD [24, 25]. While it remains controversial whether bacterial translocation is a consequence or a cause of intestinal inflammation, IBD patients have a high rate of bacterial DNA in their blood samples compared to healthy controls [25]. This can present with infectious features within a specific organ system with no local microbiological evidence of an infection. Severe Crohn’s colitis can also cause emphysematous changes within the bowel wall [26]. Since our patient had persistent symptoms for two months before seeking care, it is difficult to comment whether she developed any emphysematous changes within the bowel wall before developing EC.

In this case report we describe the first case of EC in the setting of severe Crohn’s colitis. EC has been shown to be complication of various colonic inflammatory processes. We suggest the severity of colitis in our patient was the main precipitating factor for EC. Radiological modalities can offer a non-invasive assessment of disease severity in IBD [27]. We propose that concomitant EC in decompensated IBD patients may be a marker of severe inflammation that portends poor prognosis.

## Data Availability

The datasets used and/or analysed during the current study are available from the corresponding author on reasonable request.

## Abbreviations

EC: Emphysematous cystitis
UTI: Urinary tract infection
CT: Computed tomography
MRI: Magnetic resonance imaging
CMV: Cytomegalo virus
VGH: Vancouver general hospital
IBD: Inflammatory bowel disease
PCR: Polymerase chain reaction
TB-IGRA: Tuberculosis interferon gamma release assay
HIV: Human immunodeficiency virus
IV: Intravenous
